# Supplementary material for: Instant Stress: Detection of Perceived Mental Stress Through Smartphone Photoplethysmography and Thermal Imaging
Source: JMIR Ment Health. 2019 Apr 9;6(4):e10140. doi: 10.2196/10140 (PMC6477570; doi:10.2196/10140)
Supplement: Multimedia Appendix 1 [file mental_v6i4e10140_app1.pdf]

Appendix

I. Single hidden-layer Neural Network (NN)

The single hidden-layer NN is one of the most simplistic models amongst pattern recognition frameworks which are built on the multilayer perceptron (inspired by biological systems). It has only one hidden layer and output layer. The output of node *j* in each layer can be computed with a nonlinear activation function (e.g. sigmoid, ReLU):

$$y_j(\mathrm{x}, \mathrm{w}) = f\left(\sum_{i=1}^{M} x_i w_{ji}^{(L)} + b_j^{(L)}\right). \tag{4}$$

where x_i is the input variable, *i* = 1, … , *M* (the total number of inputs), $b_j^{(L)}$ and $w_{ji}^{(L)}$ are the coefficients called *bias* and *weight*, respectively, in the $(L)^{th}$ layer of the network. The weights are updated during training. The weights are tuned with *error backpropagation* to minimize training errors.

II. k-Nearest Neighbor (kNN)

The kNN is one of the nonparametric pattern recognition algorithms to solve classification and regression problems. With a positive integer K (practically, odd number such as 1), the algorithm seeks for the K points from the training set, which are closest to a new data point x. Following this, it classifies the new point to the class which has the largest number of points within the K points.
